# Intraspinal abnormalities in MRI-evaluated congenital scoliosis: a systematic review and meta-analysis of prevalence, geographic variation, vertebral anomaly patterns, and Cobb angle

**DOI:** 10.1016/j.xnsj.2026.100943

**Published:** 2026-09-12

**Authors:** Nizar Algarni, Khalid Alrasheed, Faisal Alkhunein, Mohammed Alorayyidh, Rahaf Alrasheed, Yousef Marwan

**Affiliations:** a Department of Orthopedic Surgery, College of Medicine, King Saud University, King Khalid Road, Riyadh 11461, Saudi Arabia; b College of Medicine, King Saud University, King Khalid Road, Riyadh 11461, Saudi Arabia; c Department of Surgery, College of Medicine, Health Sciences Center, Kuwait University, Kuwait City, Kuwait

**Keywords:** Congenital scoliosis, Intraspinal abnormalities, Magnetic resonance imaging, Vertebral anomalies, Cobb angle, Systematic review, Meta-analysis

## Abstract

**Background:**

Intraspinal abnormalities are common in congenital scoliosis, yet estimates and associations with vertebral anomaly patterns and curve severity remain inconsistent. This meta-analysis aimed to estimate the pooled prevalence of intraspinal abnormalities in congenital scoliosis patients and evaluate relationships with sex, geographic region, vertebral anomaly type, and Cobb angle.

**Methods:**

PubMed, Web of Science, Scopus, and Cochrane were searched through January 1, 2026. Observational studies of congenital scoliosis patients who underwent MRI and reported intraspinal abnormalities were included. Two reviewers independently screened studies and extracted data. Methodological quality was assessed using MINORS. (PROSPERO: CRD420251276010).

**Results:**

Eighteen studies reporting 4,463 patients were included; 1,474 had intraspinal abnormalities. Overall quality was moderate. Pooled prevalence was 35% (95% CI: 29%–40%), with substantial heterogeneity (I² = 91.1%) and a prediction interval of 16%–60%. Diastematomyelia was most frequent, followed by syringomyelia and tethered cord. Within each vertebral anomaly subgroup, pooled prevalence was 26% for failure of formation, 47% for failure of segmentation, and 40% for mixed defects. In unadjusted study-level comparisons, intraspinal abnormalities were associated with higher odds of failure of segmentation (odds ratio: 1.89 [95% CI: 1.39–2.56]) and mixed abnormalities (1.43 [1.09–1.88]). Cobb angle was higher in patients with intraspinal abnormalities than in those without, but the difference was not statistically significant (mean difference: 3.30° [95% CI: −1.53 to 8.13]; p = .1805).

**Conclusions:**

Intraspinal abnormalities affect one-third of congenital scoliosis patients. Failure of segmentation and mixed vertebral anomalies may identify higher-risk subgroups, supporting MRI-based evaluation for diagnosis and surgical planning.

## Highlights

• Intraspinal abnormalities affected 35% of congenital scoliosis patients.
• Diastematomyelia was the most frequent intraspinal abnormality.
• Failure of segmentation had the highest pooled abnormality prevalence.
• Mixed vertebral anomalies were associated with higher abnormality odds.
• Cobb angle was higher with abnormalities, but not significantly.

## Introduction

Congenital scoliosis (CS) is a structural spinal deformity resulting from abnormal vertebral development during embryogenesis, accounting for a clinically significant subset of pediatric spinal deformities. It is estimated to occur in 0.5–1.0 per 1,000 live births and is commonly classified according to the underlying vertebral anomaly as failure of formation, failure of segmentation, or mixed defects [[1], [2], [3]]. These vertebral anomaly patterns are summarized in Table 1. These vertebral malformations may produce asymmetric spinal growth, and curve progression is influenced by the type and location of the anomaly, the patient’s remaining growth potential, and the magnitude of the deformity [1,2].

**Table 1:** Classification of congenital vertebral anomaly patterns [1]

Vertebral anomaly	Description	Representative examples
Failure of formation	Incomplete formation of part or all of a vertebra	Hemivertebra, wedge vertebra
Failure of segmentation	Failure of normal separation of adjacent vertebrae	Block vertebrae, unilateral longitudinal unsegmented bar
Mixed defects	Combination of vertebral formation and segmentation defects	Coexisting formation and segmentation abnormalities

Patients with CS frequently have associated abnormalities involving the intraspinal, genitourinary, cardiac, musculoskeletal, rib, gastrointestinal, and other organ systems [[2], [3], [4], [5]]. Given the close embryologic relationship between vertebral and neural development, intraspinal abnormalities represent a particularly important group of associated abnormalities in this population. Commonly detected abnormalities include diastematomyelia, syringomyelia, tethered cord, low-lying conus medullaris, Chiari malformation, fatty filum, and intraspinal masses [5,6]. Although some intraspinal abnormalities may be clinically silent, their recognition is important because certain lesions, such as split cord malformation and tethered cord, may require neurosurgical evaluation or influence the timing and planning of deformity correction [1,5]. However, not all MRI-detected abnormalities require intervention before scoliosis surgery, and management depends on the specific abnormality, neurological status, and clinical context [5]. Therefore, whole-spine magnetic resonance imaging (MRI) is widely used in the diagnostic and preoperative evaluation of patients with CS, particularly before corrective surgery [5,7]. The present review is intended primarily to inform MRI evaluation and risk stratification in referral and preoperative settings, particularly before deformity surgery.

The reported prevalence of intraspinal abnormalities in CS varies substantially across studies, with prior studies reporting estimates from 15% to more than 50% [[5], [6], [7], [8], [9], [10]]. This variability likely reflects differences in cohort composition, including surgical versus nonsurgical populations, patient demographics, imaging practices, definitions of intraspinal abnormalities, geographic setting, and study design. A previous systematic review and meta-analysis by Wang et al. included 10 studies published up to 2019 and reported that 671 of 1,863 patients with CS who underwent MRI had intraspinal abnormalities, corresponding to a pooled estimate of 37%. In that review, diastematomyelia, syringomyelia, and tethered cord were the most frequently reported intraspinal abnormalities [6].

Since that prior review, additional studies have provided larger samples, broader geographic representation, more detailed reporting of vertebral anomaly patterns, and radiographic parameters such as Cobb angle [3,5,[11], [12], [13]]. Recent data suggest that intraspinal abnormalities may be more common in patients with failure of segmentation or mixed vertebral deformities than in those with failure of formation, and some cohorts have reported larger Cobb angles among patients with intraspinal abnormalities [3,10]. However, the overall relationship between intraspinal abnormalities, vertebral anomaly type, and curve severity remains uncertain across studies. Given that the overall prevalence of intraspinal abnormalities has already been estimated in a prior meta-analysis, an important remaining question is whether risk differs according to vertebral anomaly pattern and Cobb angle. Clarifying these differences may help identify higher-risk subgroups and refine MRI-based clinical risk stratification.

Therefore, an updated systematic review and meta-analysis is warranted. The present study aimed to estimate the pooled prevalence of intraspinal abnormalities in patients with CS and to evaluate associations with sex, geographic region, vertebral anomaly pattern, and Cobb angle.

## Methods and materials

A systematic review and meta-analysis was prospectively registered in the International Prospective Register of Systematic Reviews (PROSPERO) (registration number: CRD420251276010). This study adheres to the Preferred Reporting Items for Systematic Reviews and Meta-Analyses (PRISMA 2020) guidelines [14].

### Data source and search strategy

A systematic search of MEDLINE via PubMed, Web of Science, Scopus, and Cochrane was performed from inception until January 1, 2026. The search combined the following terms: (``MRI'' OR ``magnetic'' OR ``resonance'' OR ``imaging'') AND ``congenital'' AND ``scoliosis.'' Full search strategies for each database are presented in Supplementary Table 1.

### Eligibility criteria

Studies were included if they enrolled patients with a confirmed diagnosis of CS. Eligible studies were required to assess specific intraspinal abnormalities using MRI and to provide sufficient data to calculate the prevalence of these abnormalities. Some included studies used routine or consecutive MRI evaluation, whereas others reported MRI-evaluated subsets. MRI indications were not consistently reported. Intraspinal abnormalities were defined as MRI-detected abnormalities of the spinal cord and associated neural-axis structures reported by the included studies, including diastematomyelia, syringomyelia, tethered cord, low-lying conus, Chiari malformation, fatty filum, intraspinal masses or tumors, spinal cysts, dural ectasia, spina bifida (excluding occulta), and other spinal cord abnormalities. Definitions and reporting criteria were based on those used in the original studies. Reported terminology was harmonized to these predefined diagnostic categories when terminology could be mapped unambiguously. Source-specific or ambiguous categories that could not be reassigned without reinterpretation were retained separately. Observational studies, including cross-sectional studies, prospective or retrospective cohort studies, and case-control studies, were considered eligible if they were published as English-language, peer-reviewed full-text articles. Studies were excluded if they focused on non-CS, including idiopathic, neuromuscular, or syndromic scoliosis, unless data for CS patients were reported separately. Case reports, small case series, reviews, editorials, conference abstracts, and animal or cadaveric studies were excluded.

### Study selection and data extraction

After duplicate removal, 2 authors independently screened all titles and abstracts of retrieved records, followed by full-text review of potentially eligible reports. Disagreements were resolved by discussion or consultation with a third author. For overlapping reports, data were extracted from the most complete dataset to avoid double-counting. The 2 Lin et al. publications [11,12] were based on the same 1,289-patient surgical cohort and were therefore treated as a single study. Quantitative data used in the meta-analyses were extracted from Lin et al. [12], while Lin et al. [11] provided complementary information on vertebral anomaly location. The cohort was included only once in each pooled analysis. Data extraction was then performed independently by 2 authors using a standardized spreadsheet. Extracted variables included the first author's name, publication year, country, total CS patients sample size, number of CS patients with intraspinal abnormalities, age, sex, CS subtypes, specific intraspinal abnormality subtypes, and Cobb angle. When available, group-specific data were extracted for patients with and without intraspinal abnormalities, including CS subtype counts and Cobb angle means, standard deviations, or ranges. Geographic region and publication era were derived from country and publication year for subgroup analyses.

### Methodological quality assessment

The methodological quality of included studies was assessed independently by 2 authors using the Methodological Index for Non-Randomized Studies (MINORS) [15]. Noncomparative studies were scored across 8 items, with a maximum score of 16, while comparative studies were scored across 12 items, with a maximum score of 24. Each item was scored on a scale of 0 to 2. For noncomparative studies, scores of ≤8, 9–14, and 15–16 were considered poor, moderate, and good quality, respectively. For comparative studies, scores of ≤14, 15–22, and 23–24 were considered poor, moderate, and good quality, respectively. Disagreements were resolved by consensus or consultation with a third author.

### Statistical analysis

All meta-analyses were performed using R version 4.6.0 (R Foundation for Statistical Computing, Vienna, Austria), with the meta package. Pooled prevalence estimates were calculated using random-effects models with logit-transformed proportions, inverse-variance weighting, and restricted maximum-likelihood estimation of between-study variance. Study-specific confidence intervals for proportions were calculated using the Clopper–Pearson exact binomial method, and pooled estimates were back-transformed to proportions for reporting. Results were reported as proportions with 95% confidence intervals (CIs) and prediction intervals. Analyses were performed for the overall prevalence of intraspinal abnormalities, sex-specific prevalence, CS subtype-specific prevalence, and specific intraspinal abnormality subtypes. Subgroup analyses were conducted by geographic region, publication era, and sex for the overall prevalence of intraspinal abnormalities. Comparisons of CS subtypes between patients with and without intraspinal abnormalities were analyzed using random-effects Mantel–Haenszel odds ratios (ORs). Two-by-two tables were checked for zero-event cells. When zero-event cells were present, a continuity correction of 0.5 was applied. Cobb angle differences were analyzed using random-effects mean differences (MDs) when group-specific sample size, mean, and standard deviation were available. Heterogeneity was assessed using Cochran’s Q test, I², and τ². Leave-one-out sensitivity analyses were performed to evaluate the influence of individual studies, and funnel plots were used to assess small-study effects when sufficient studies were available. Statistical significance was defined as p < .05.

## Results

### Study selection

The initial literature search resulted in 4,868 records. After removing 1,216 duplicate records, 3,652 studies were screened. Of these, 3,606 were excluded, leaving 46 full-text reports for full-text review. Eighteen studies met the inclusion criteria and were included in the systematic review and meta-analysis, as shown in Fig. 1.

**Fig. 1:**
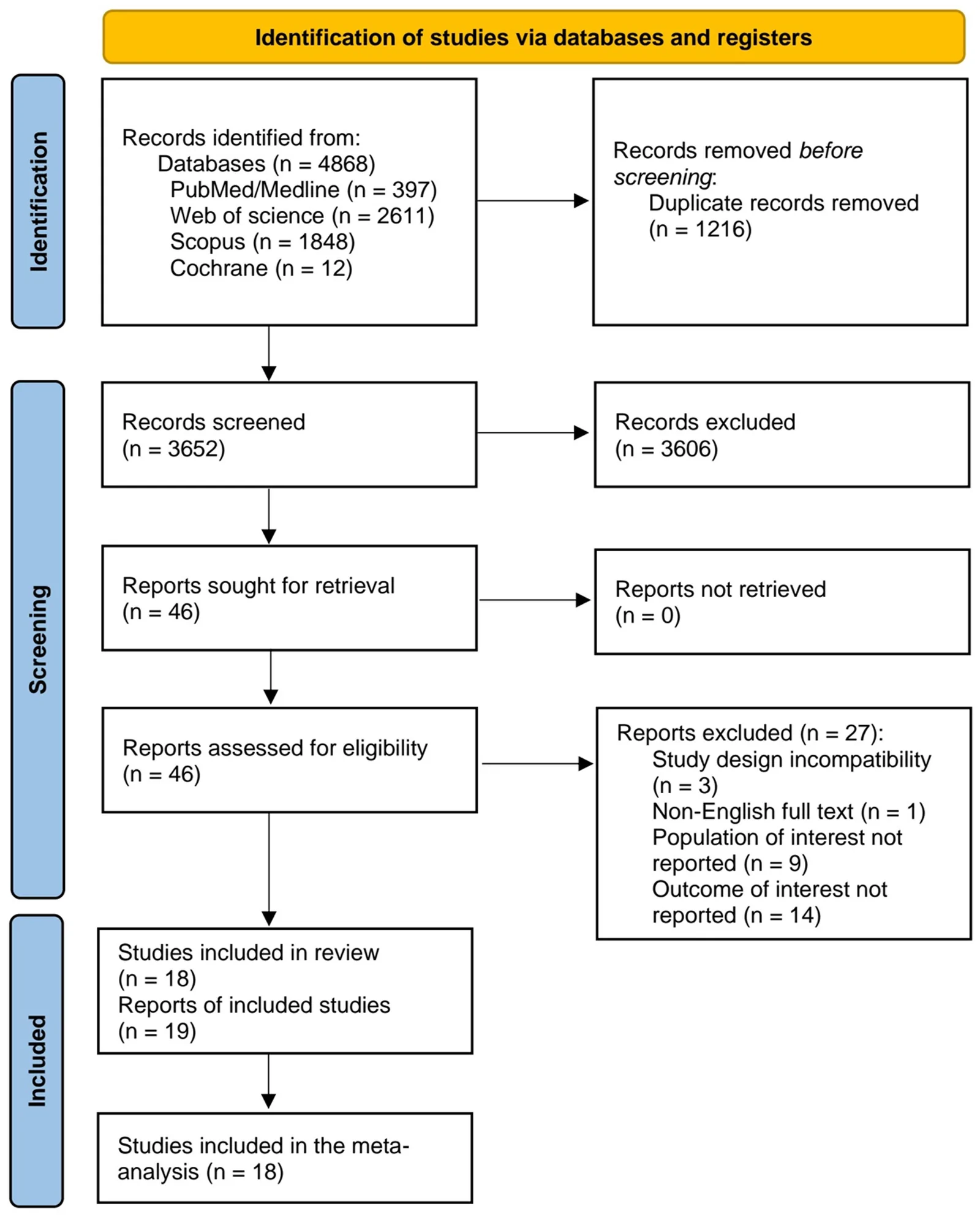
PRISMA flow diagram.

### Characteristics of included studies

Table 2 summarizes 18 studies published from 2001 to 2026, which included 4,463 patients with CS. The included studies were conducted in China (27.8%), India (22.2%), Iran (16.7%), the USA (16.7%), Saudi Arabia (5.6%), Pakistan (5.6%), and Turkey (5.6%). Overall, 1,474 patients had intraspinal abnormalities. Most cohorts were pediatric, with mean ages generally ranging from 4.9 to 14.7 years among studies reporting age.

**Table 2:** Characteristics of included studies

Study	Country	CS with intraspinal abnormalities /CS (n/N)	Mean age ± SD (range), y	Males with abnormalities (n/N)	Females with abnormalities (n/N)	CS types: Failure of formation with abnormalities (n/N)	CS types: Failure of segmentation with abnormalities (n/N)	CS types: Mixed with abnormalities (n/N)	MINORS
Alkhunein et al. [19]	Saudi Arabia	6/25	NA	4/11	2/14	NA	NA	NA	9
Khan et al. [20]	Pakistan	25/122	14.7 ± 7.4 (4–54)	NA	NA	NA	NA	NA	10
Johari et al. [13]	India	123/229	7.1 ± 4.6 (0.4–19)	44/88	79/141	45/114	28/39	50/76	8
Wu et al. [3]	China	186/614	6.4 ± 6.3	68/281	118/333	24/194	44/89	118/331	10
Lin et al. (I/II) [11,12]	China	379/1289	12.4 ± 8.1	125/593	254/696	101/633	114/251	164/405	10
Yarandi et al. [21]	Iran	15/34	11.6 ± 5.7 (3–28)	NA	NA	NA	NA	NA	10
Mohanty et al. [5]	India	48/227	8.8 ± 5.2	NA	NA	25/144	15/38	8/45	9
Furdock et al. [4]	USA	113/267	7.3 ± 3 (0.003–18)	NA	NA	NA	NA	NA	8
Sevencan et al. [9]	Turkey	124/231	12.6 ± 7.1 (0.1–17)	43/94	81/137	65/129	41/64	18/38	8
Hou et al. [22]	China	54/120	12.3 (2–45)	NA	NA	NA	NA	NA	15
Gupta et al. [17]	India	56/119	5.3 ± NR (<1–26)	16/44	40/75	21/61	15/23	20/35	9
Ghandhari et al. [23]	Iran	44/202	13.5 ± 6.6 (2–38)	16/90	28/112	28/108	10/64	6/30	15
Shen et al. [10]	China	99/226	13.9 (2–21)	33/94	66/132	27/96	29/48	43/82	7
Liu et al. [24]	China	132/539	12.8 ± 5.8	37/214	95/325	71/301	45/168	16/70	7
Rajasekaran et al. [7]	India	21/60	5.2	8/19	13/41	NA	NA	NA	9
Erfani et al. [25]	Iran	19/46	NA	NA	NA	NA	NA	NA	8
Belmont et al. [26]	USA	18/76	4.9	NA	NA	NA	NA	NA	9
Suh et al. [16]	USA	12/37	NA	6/17	6/20	5/19	1/4	6/14	8

USA, United States; CS, congenital scoliosis; NA, not available.

### Methodological quality assessment

For the 16 noncomparative studies, the average MINORS score was 8.7 (range, 7–10), suggesting overall moderate quality, although several studies fell within the poor-quality range. The 2 comparative studies both had scores of 15, suggesting moderate quality (Table 2). The most common methodological limitations were retrospective data collection, absence of prospective sample-size calculation, incomplete reporting of loss to follow-up, and limited reporting of unbiased outcome assessment (Supplementary Table 2).

### Overall prevalence of intraspinal abnormalities

The pooled prevalence of intraspinal abnormalities was 35% across 18 studies involving 4,463 patients (95% CI, 29%–40%). Heterogeneity was substantial (I² = 91.1%, p < .0001), with a prediction interval of 16%–60%. Leave-one-out analysis showed stable pooled estimates. Visual inspection of the funnel plot revealed no obvious asymmetry, although the assessment was limited (Fig. 2).

**Fig. 2:**
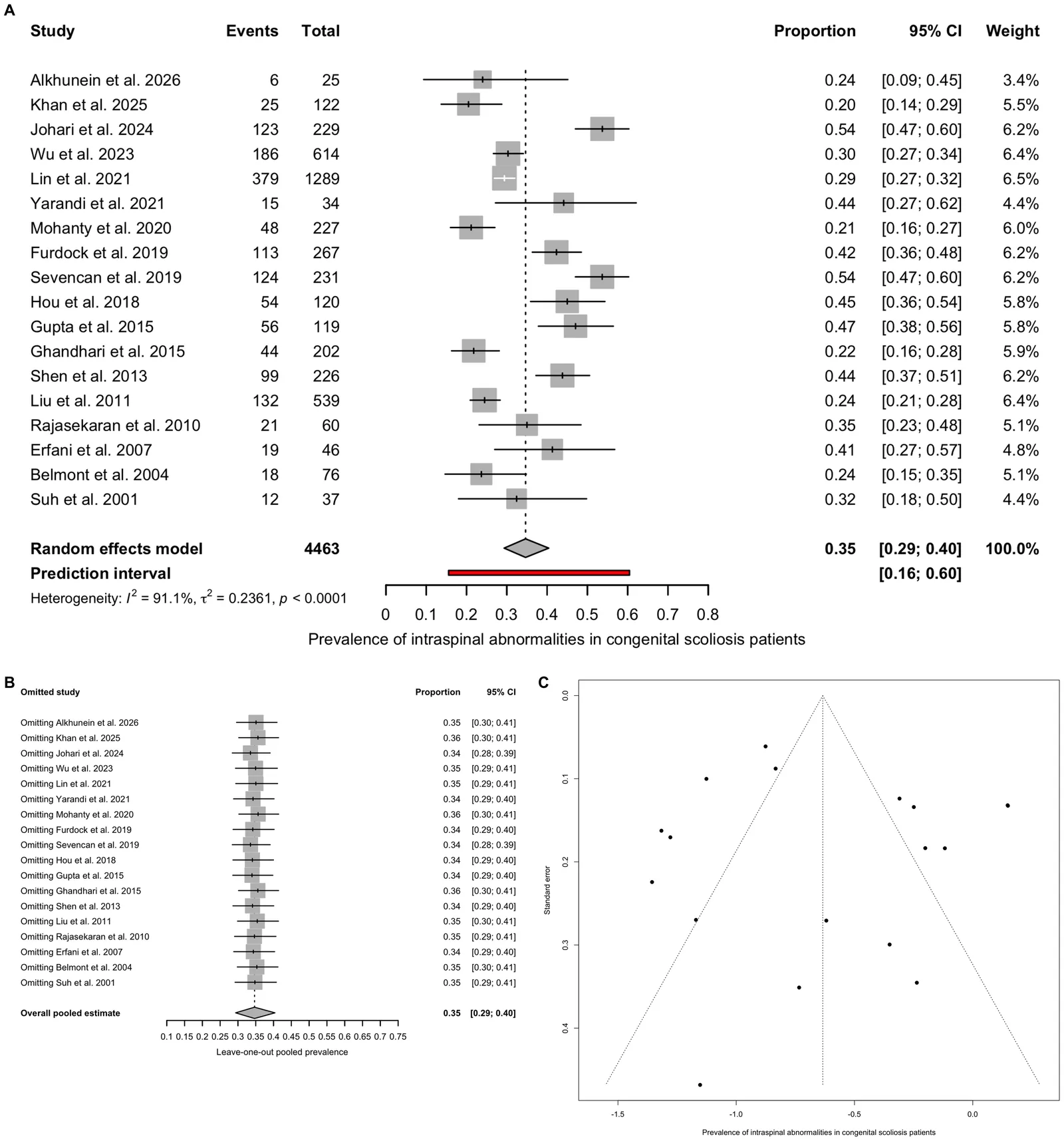
Pooled prevalence of intraspinal abnormalities in congenital scoliosis patients across 18 studies comprising 4,463 patients. (A) Forest plot. (B) Leave-one-out sensitivity analysis. (C) Funnel plot. CI, confidence interval.

### Subgroup analyses by geographic region and publication era

Subgroup analysis showed similar pooled prevalence across regions: East Asia 34%, South Asia 34%, Middle East 37%, and North America 33%, with no significant regional subgroup difference (p = .9820). Stratification by publication era also showed no significant difference between studies published in ≤2019 and 2020–2026 (37% vs. 31%; p = .3310). Overall heterogeneity remained substantial (I² = 91.1%, p < .0001) (Fig. 3).

**Fig. 3:**
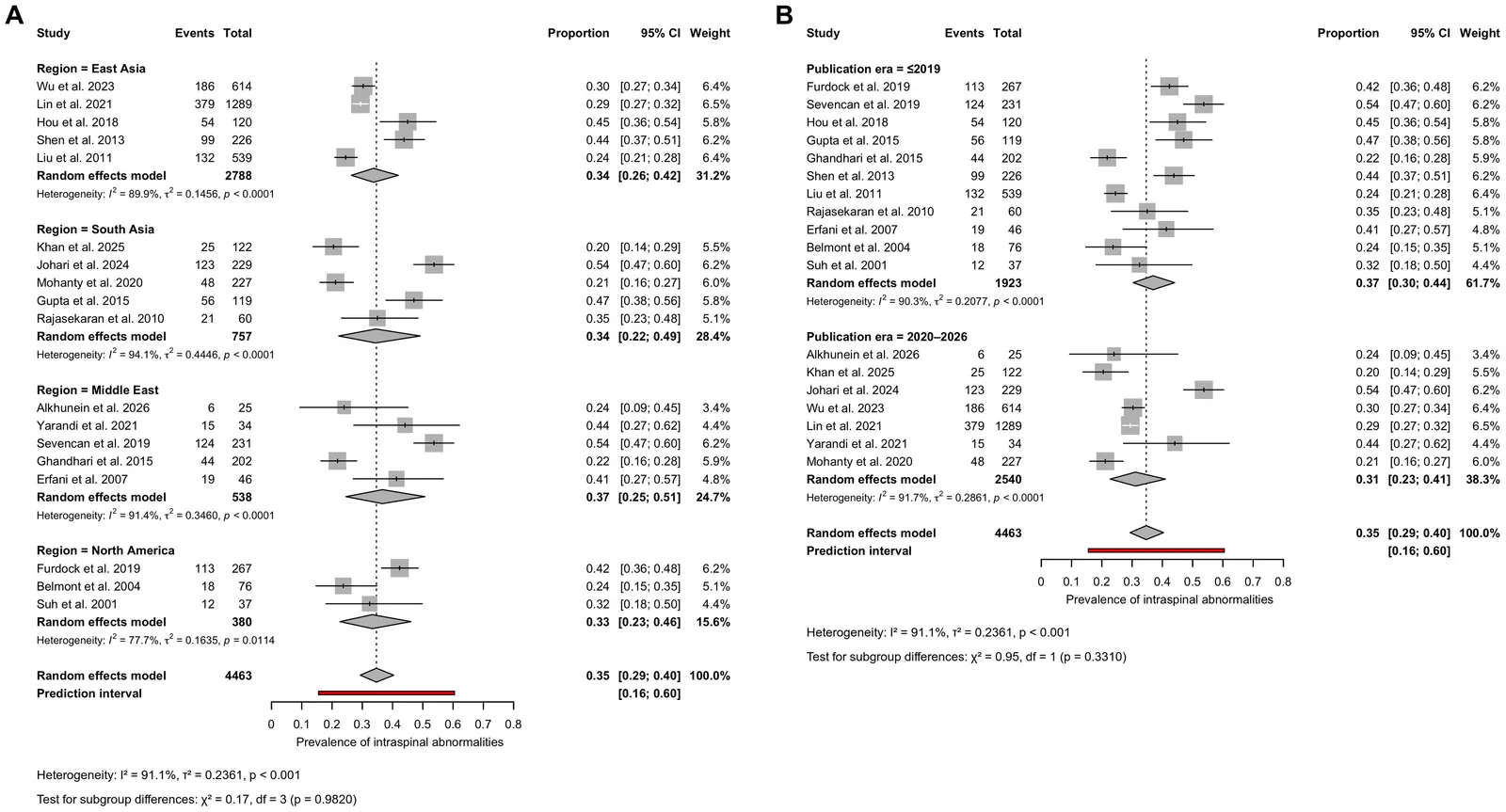
Subgroup analyses of the pooled prevalence of intraspinal abnormalities in patients with congenital scoliosis. (A) Forest plot stratified by geographic region: East Asia, 5 studies and 2,788 patients; South Asia, 5 studies and 757 patients; Middle East, 5 studies and 538 patients; North America, 3 studies and 380 patients. (B) Forest plot stratified by publication era: ≤2019, 11 studies and 1,923 patients; 2020–2026, 7 studies and 2,540 patients. CI, confidence interval; df, degrees of freedom.

### Sex-specific prevalence of intraspinal abnormalities

Among male patients, the pooled prevalence of intraspinal abnormalities was 31% (95% CI, 24%–39%). Heterogeneity was substantial (I² = 85.6%, p < .0001), with a wide prediction interval (12%–60%). Leave-one-out analysis showed stable pooled estimates, while funnel plot partial asymmetry suggested possible small-study effects (Fig. 4). Among female patients, the pooled prevalence of intraspinal abnormalities was 39% (95% CI, 32%–48%). Heterogeneity was substantial (I² = 87.7%, p < .0001), with a prediction interval of 16%–68%. Leave-one-out analysis showed stable estimates, while the funnel plot partial asymmetry suggested possible small-study effects (Fig. 5). In the sex subgroup analysis, the pooled prevalence was higher among female patients than male patients. However, the difference between subgroups was not statistically significant (p = .13) (Fig. 6).

**Fig. 4:**
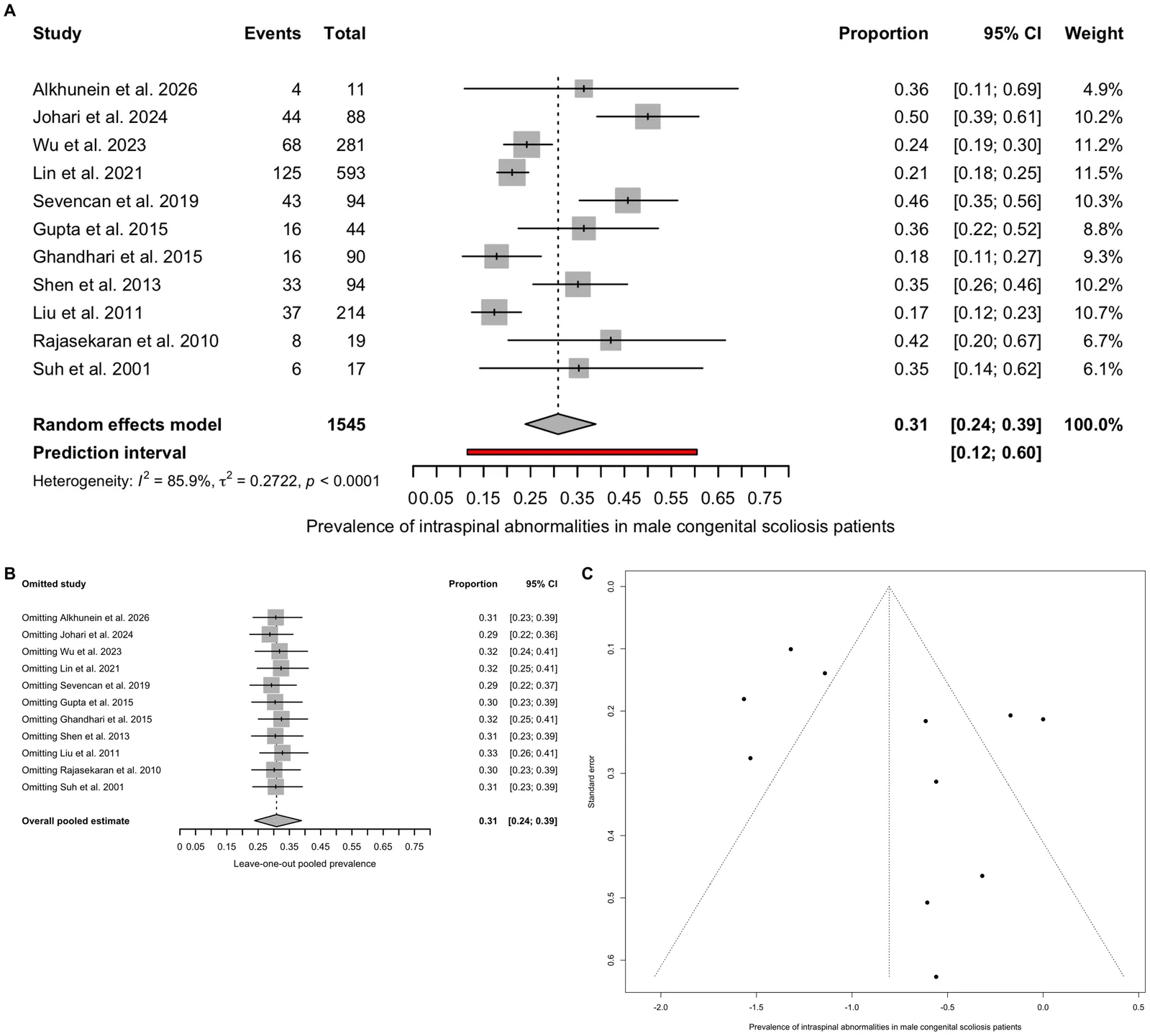
Pooled prevalence of intraspinal abnormalities in male congenital scoliosis patients across 11 studies comprising 1,545 male patients. (A) Forest plot. (B) Leave-one-out sensitivity analysis. (C) Funnel plot. CI, confidence interval.

**Fig. 5:**
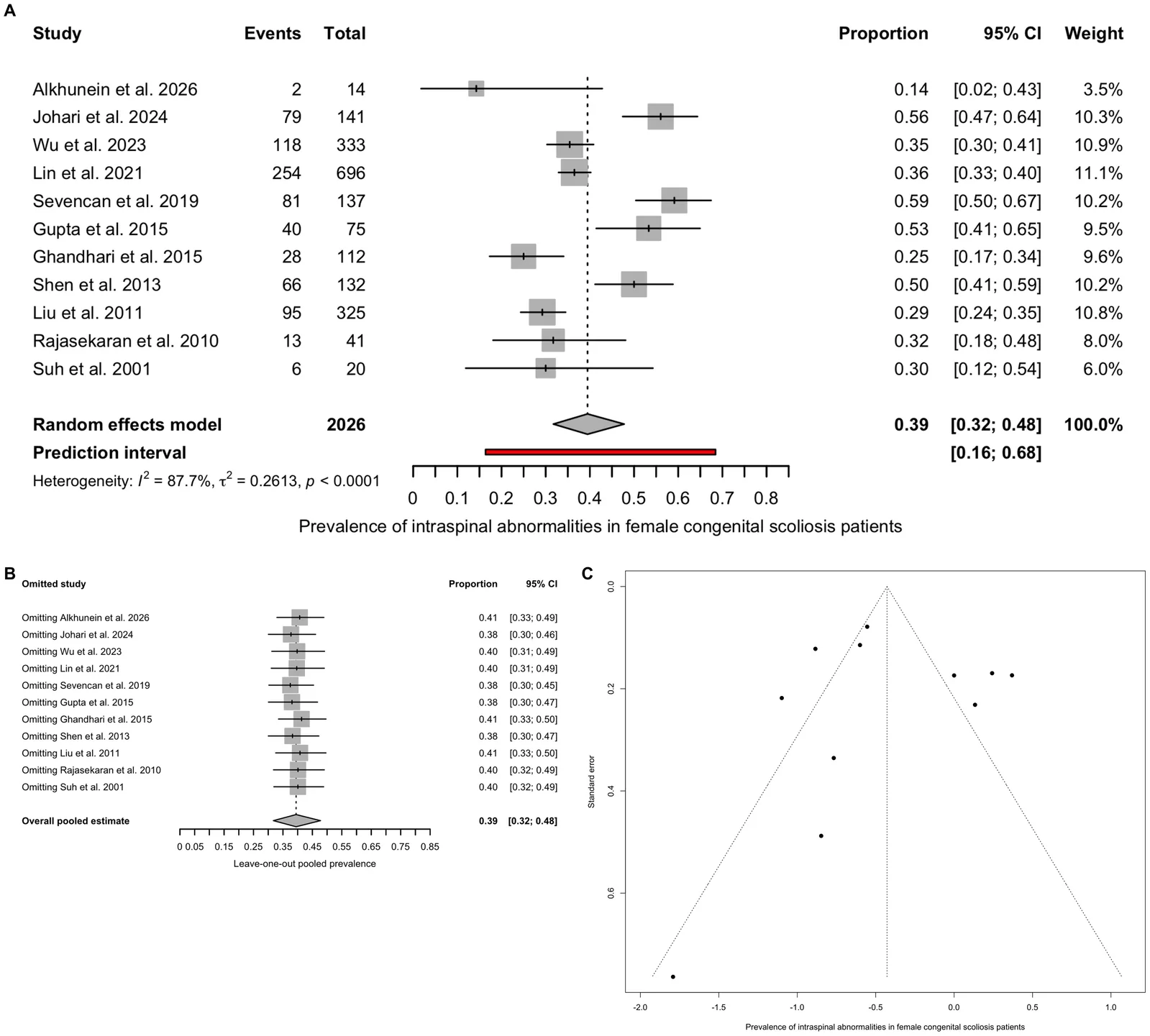
Pooled prevalence of intraspinal abnormalities in female congenital scoliosis patients across 11 studies comprising 2,026 female patients. (A) Forest plot. (B) Leave-one-out sensitivity analysis. (C) Funnel plot. CI, confidence interval.

**Fig. 6:**
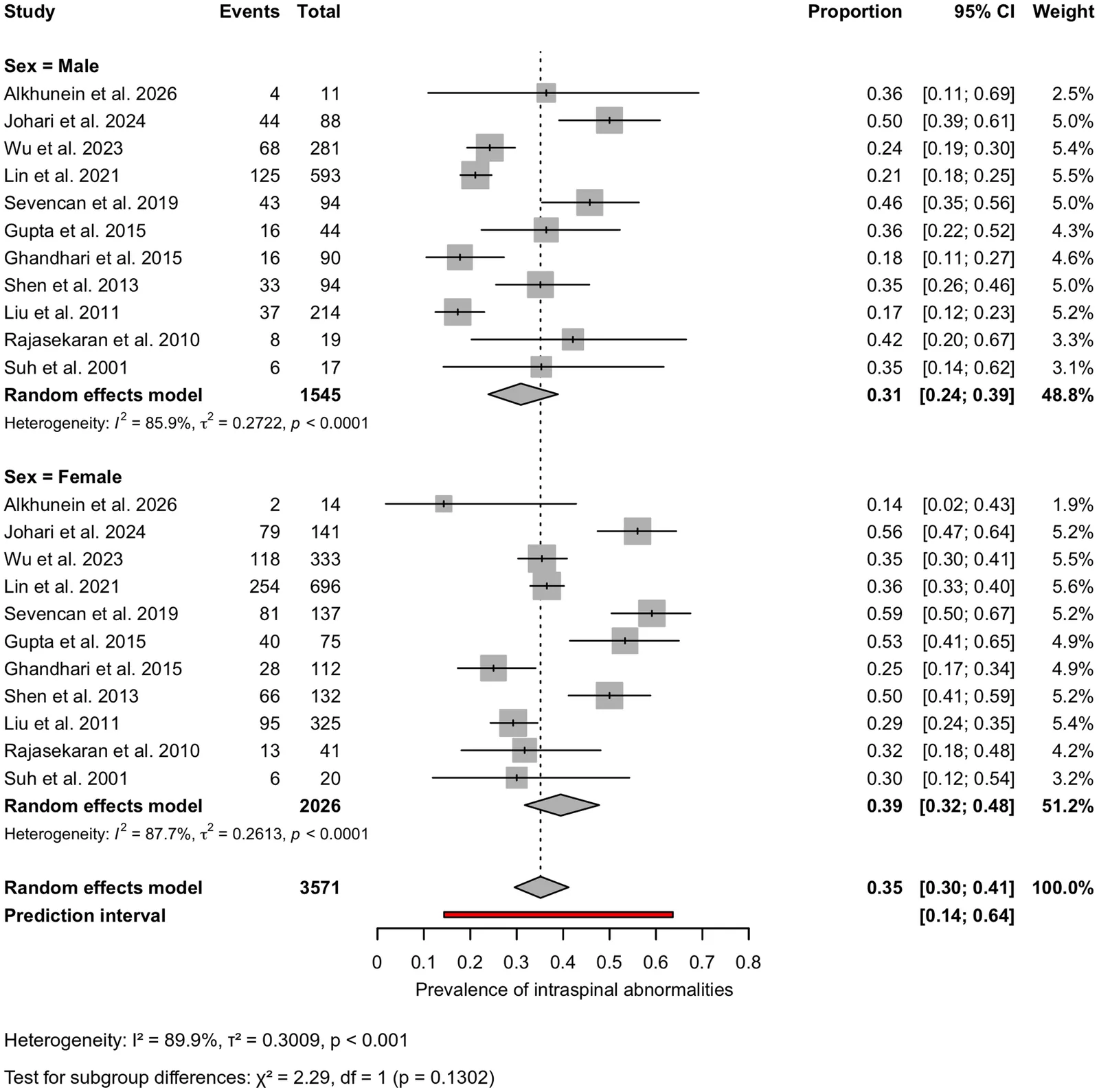
Sex subgroup analysis of the pooled prevalence of intraspinal abnormalities in congenital scoliosis patients across 11 studies comprising 3,571 patients with sex-specific data, including 1,545 male and 2,026 female patients. CI, confidence interval; df, degrees of freedom.

### Distribution of intraspinal abnormalities

Among 1,474 patients with intraspinal abnormalities, the most common abnormality was diastematomyelia (53.5%), followed by syringomyelia (40.4%) and tethered cord (29.9%). Less frequently reported abnormalities included low conus (12.6%), Chiari malformation (7.1%), intraspinal mass (5.0%), fatty filum (2.6%), spina bifida excluding occulta (2.3%), and cyst (1.7%) (Table 3).

**Table 3:** Intraspinal abnormalities

Intraspinal abnormality	Number of patients	Percent (%)
Diastematomyelia	788	53.5
Syringomyelia	595	40.4
Tethered cord	440	29.9
Low conus	186	12.6
Chiari malformation	104	7.1
Intraspinal mass	74	5.0
Fatty filum	39	2.6
Spina bifida (occulta excluded)	34	2.3
Cyst	25	1.7
Dural ectasia	5	0.3
Syringomyelus	4	0.3
Tumor	4	0.3
Undiagnosed cord MRI hyperintensity	1	0.1

Patients could have more than 1 intraspinal abnormality; therefore, percentages are not mutually exclusive and may sum to more than 100%. Syringomyelus was retained as reported in the source study.

### Cobb angle and intraspinal abnormalities

Table 4 summarizes Cobb angle reporting across the included studies. Among the 18 included studies, 1,474 patients were reported to have intraspinal abnormalities, while 2,989 patients had no intraspinal abnormalities. The overall mean Cobb angle was reported in 9 studies and ranged from 35.4° to 71.2°. Six studies provided Cobb angle data separately for patients with and without intraspinal abnormalities; mean Cobb angles ranged from 31.4° to 71.4° in patients with intraspinal abnormalities and from 37.3° to 71.2° in those without abnormalities. Suh et al. [16] reported group-specific means without standard deviations and was therefore included descriptively but excluded from the pooled analysis.

**Table 4:** Comparison of Cobb angle between congenital scoliosis patients with and without intraspinal abnormalities

Study	Total mean Cobb angle ± SD (range), °	Intraspinal abnormalities (n)	Mean Cobb angle with intraspinal abnormalities ± SD (range), °	No intraspinal abnormalities (n)	Mean Cobb angle without intraspinal abnormalities ± SD (range), °	Included in quantitative synthesis
Alkhunein et al. [19]	NA	6	NA	19	NA	No
Khan et al. [20]	65.4 ± 14.8 (30–110)	25	NA	97	NA	No
Johari et al. [13]	46.2 ± 22.4 (5–145)	123	49.4 ± 23.8 (8–145)	106	42.5 ± 20.1 (5–97)	Yes
Wu et al. [3]	60.8 ± 26.5	186	68.3 ± 26.2	428	57.8 ± 26	Yes
Lin et al. (I/II) [11,12]	NA	379	NA	910	NA	No
Yarandi et al. [21]	55.3 ± 22.8 (10–160)	15	NA	19	NA	No
Mohanty et al. [5]	41 ± 3.2	48	40.9 ± 2.3	179	41.1 ± 3.4	Yes
Furdock et al. [4]	NA	113	NA	154	NA	No
Sevencan et al. [9]	NA	124	NA	107	NA	No
Hou et al. [22]	NA	54	NA	66	NA	No
Gupta et al. [17]	NA	56	NA	63	NA	No
Ghandhari et al. [23]	71.2 ± 24.9 (30–140)	44	71.4 ± 22.9	158	71.2 ± 25.3	Yes
Shen et al. [10]	66.7	99	NA	127	NA	No
Liu et al. [24]	NA	132	NA	407	NA	No
Rajasekaran et al. [7]	46.3 ± 13.3 (27–89)	21	45 ± 15.5 (27–89)	39	47 ± 12 (29–77)	Yes
Erfani et al. [25]	NA	19	NA	27	NA	No
Belmont et al. [26]	NA	18	NA	58	NA	No
Suh et al. [16]	35.4	12	31.4	25	37.3	No*

NA, not available.

⁎ Suh et al. [16] reported group-specific means without standard deviations and was therefore excluded from the quantitative synthesis.

Across 5 studies, Cobb angle did not differ significantly between patients with and without intraspinal abnormalities. The pooled mean difference was 3.30° higher in patients with intraspinal abnormalities, but this was not statistically significant (95% CI, −1.53 to 8.13; p = .1805). Heterogeneity was substantial (I² = 85.0%, p < .0001), with a wide prediction interval (−11.56 to 18.15). Leave-one-out analysis showed stable pooled estimates with no statistically significant change after removing any single study, while funnel plot interpretation was limited by the small number of included studies (Fig. 7).

**Fig. 7:**
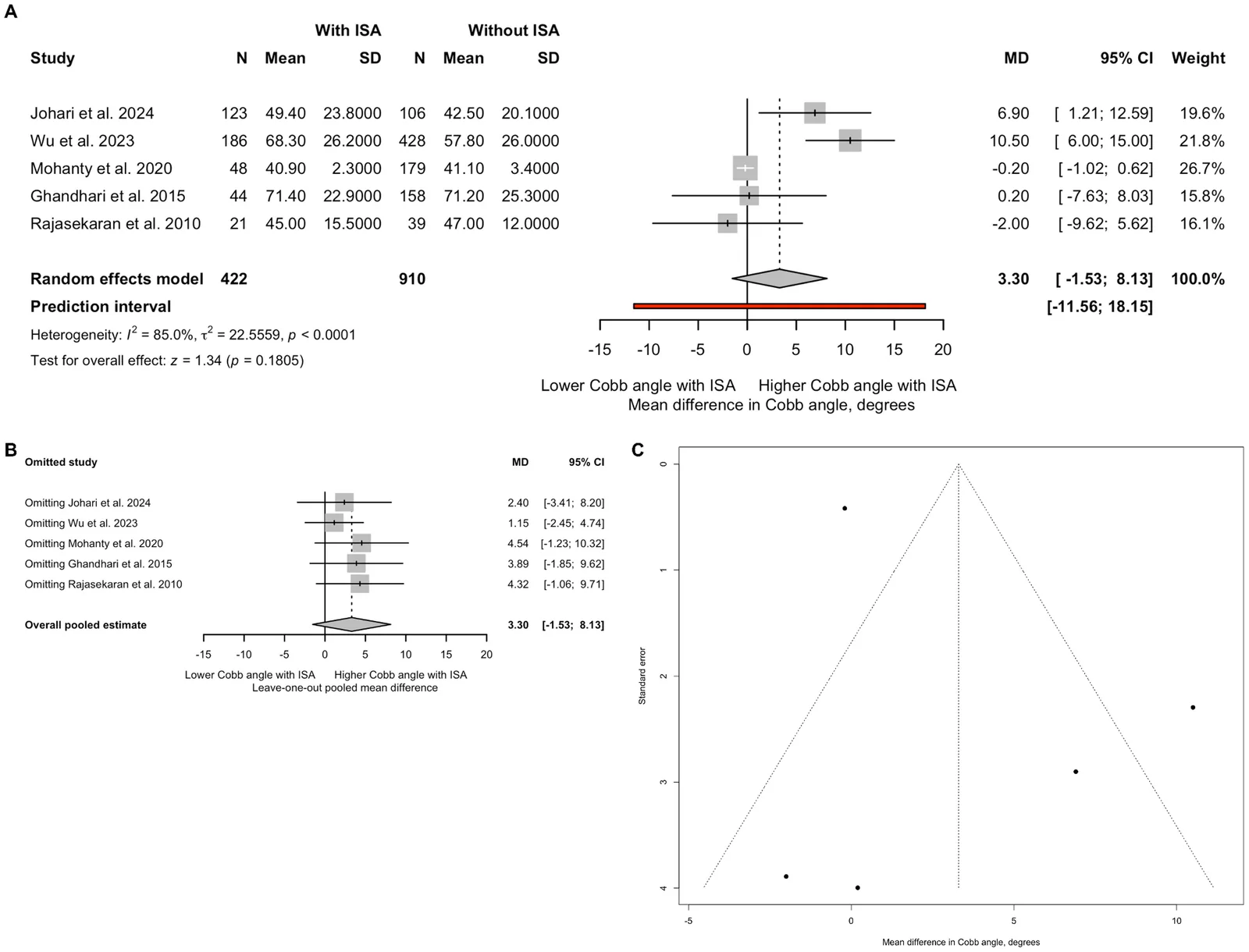
Cobb angle comparison between congenital scoliosis patients with and without intraspinal abnormalities across 5 studies comprising 1,332 patients, including 422 with and 910 without intraspinal abnormalities. (A) Forest plot comparing mean Cobb angle between patients with and without intraspinal abnormalities. (B) Leave-one-out sensitivity analysis. (C) Funnel plot. CI, confidence interval; ISA, intraspinal abnormalities; MD, mean difference; SD, standard deviation.

### Prevalence of intraspinal abnormalities in patients with different CS types

By CS subtype, the pooled prevalence of intraspinal abnormalities was lowest in patients with failure of formation at 26% (95% CI, 19%–34%; seen in Fig. 8), and higher in those with failure of segmentation at 47% (95% CI, 34%–60%; seen in Fig. 9) and mixed abnormalities at 40% (95% CI, 30%–51%; seen in Fig. 10). Substantial heterogeneity was observed across all subgroups (I² = 83.0%–91.2%, all p < .0001), with wide prediction intervals, indicating considerable between-study variability. Leave-one-out analyses showed generally stable pooled estimates, suggesting that no single study substantially influenced the results.

**Fig. 8:**
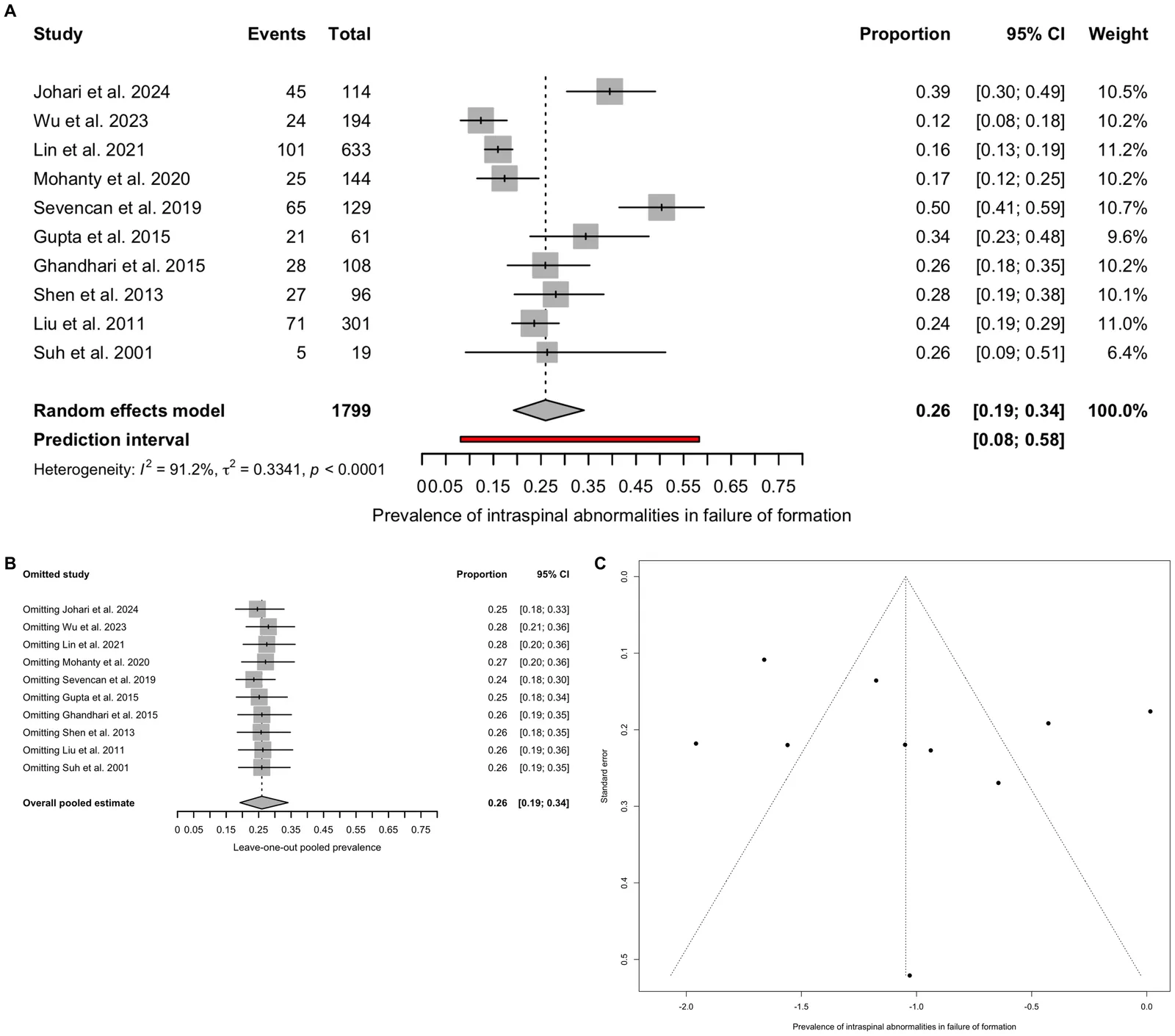
Pooled prevalence of intraspinal abnormalities in congenital scoliosis patients with failure of formation across 10 studies comprising 1,799 patients. (A) Forest plot. (B) Leave-one-out sensitivity analysis. (C) Funnel plot. CI, confidence interval.

**Fig. 9:**
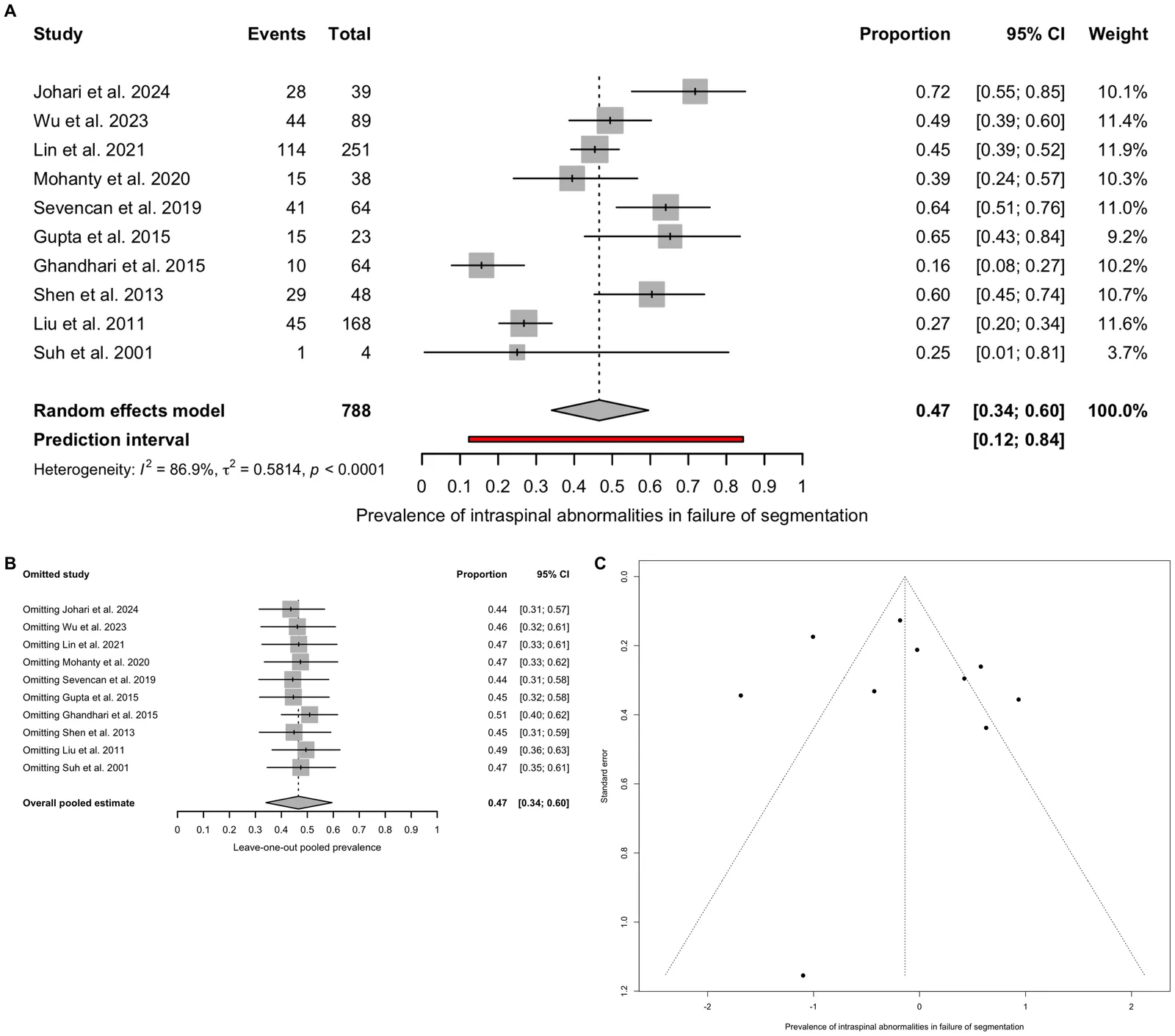
Pooled prevalence of intraspinal abnormalities in congenital scoliosis patients with failure of segmentation across 10 studies comprising 788 patients. (A) Forest plot. (B) Leave-one-out sensitivity analysis. (C) Funnel plot. CI, confidence interval.

**Fig. 10:**
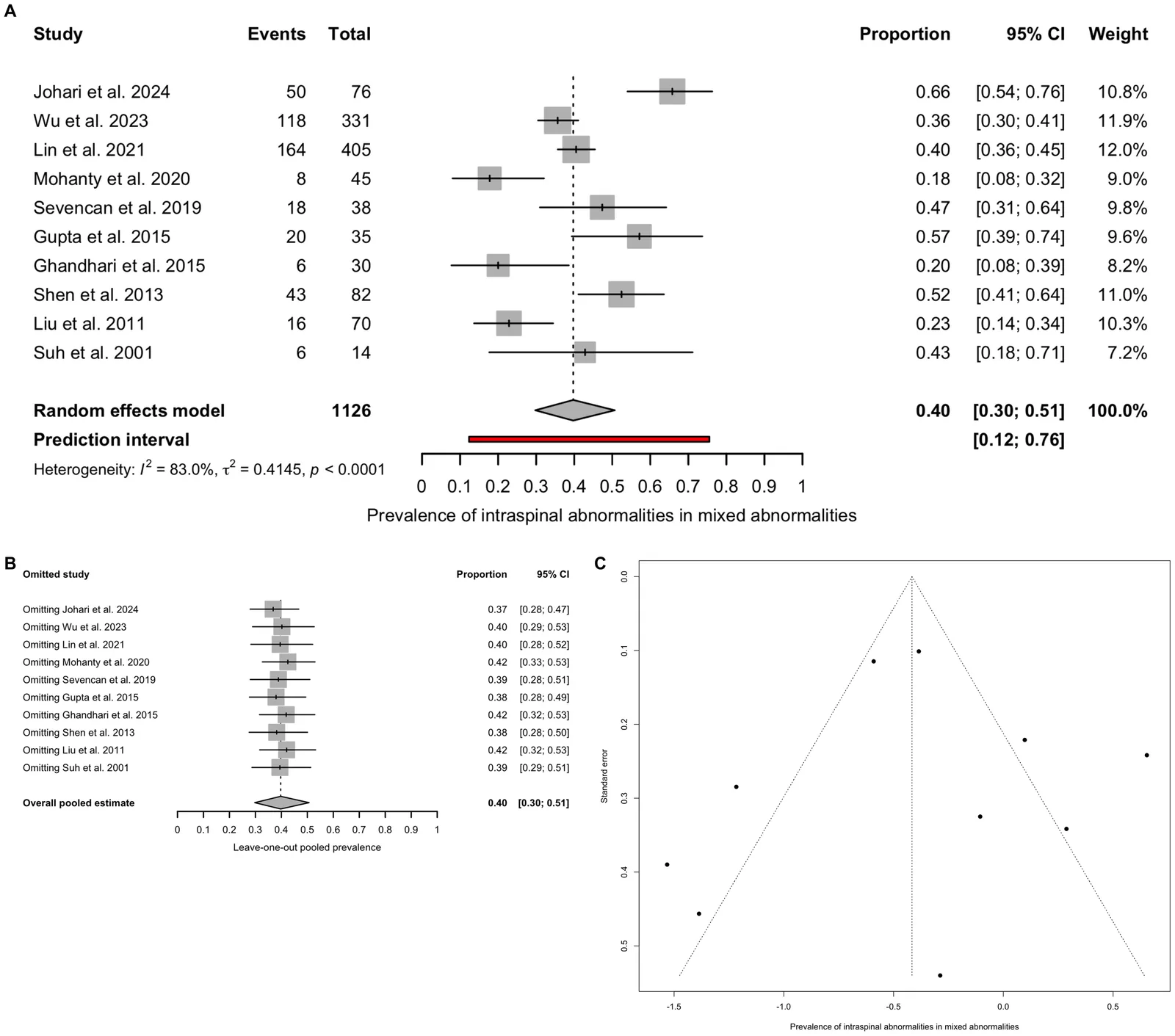
Pooled prevalence of intraspinal abnormalities in congenital scoliosis patients with mixed abnormalities across 10 studies comprising 1,126 patients. (A) Forest plot. (B) Leave-one-out sensitivity analysis. (C) Funnel plot. CI, confidence interval.

### Association between CS types and intraspinal abnormalities

All 3 vertebral-type comparisons were based on the same 10 studies comprising 3,713 patients. Compared with patients without intraspinal abnormalities, patients with intraspinal abnormalities had significantly lower odds of failure of formation (OR = 0.47; 95% CI, 0.31–0.71; p = .0003; seen in Fig. 11) and significantly higher odds of failure of segmentation (OR = 1.89; 95% CI, 1.39–2.56; p < .0001; seen in Fig. 12) and mixed abnormalities (OR = 1.43; 95% CI, 1.09–1.88; p = .0092; seen in Fig. 13). These estimates are interdependent because they compare categories within the same vertebral anomaly classification and were derived from the same cohorts. Heterogeneity ranged from moderate to substantial (I² = 51.7%–84.4%), and leave-one-out analyses showed stable pooled estimates across all 3 comparisons.

**Fig. 11:**
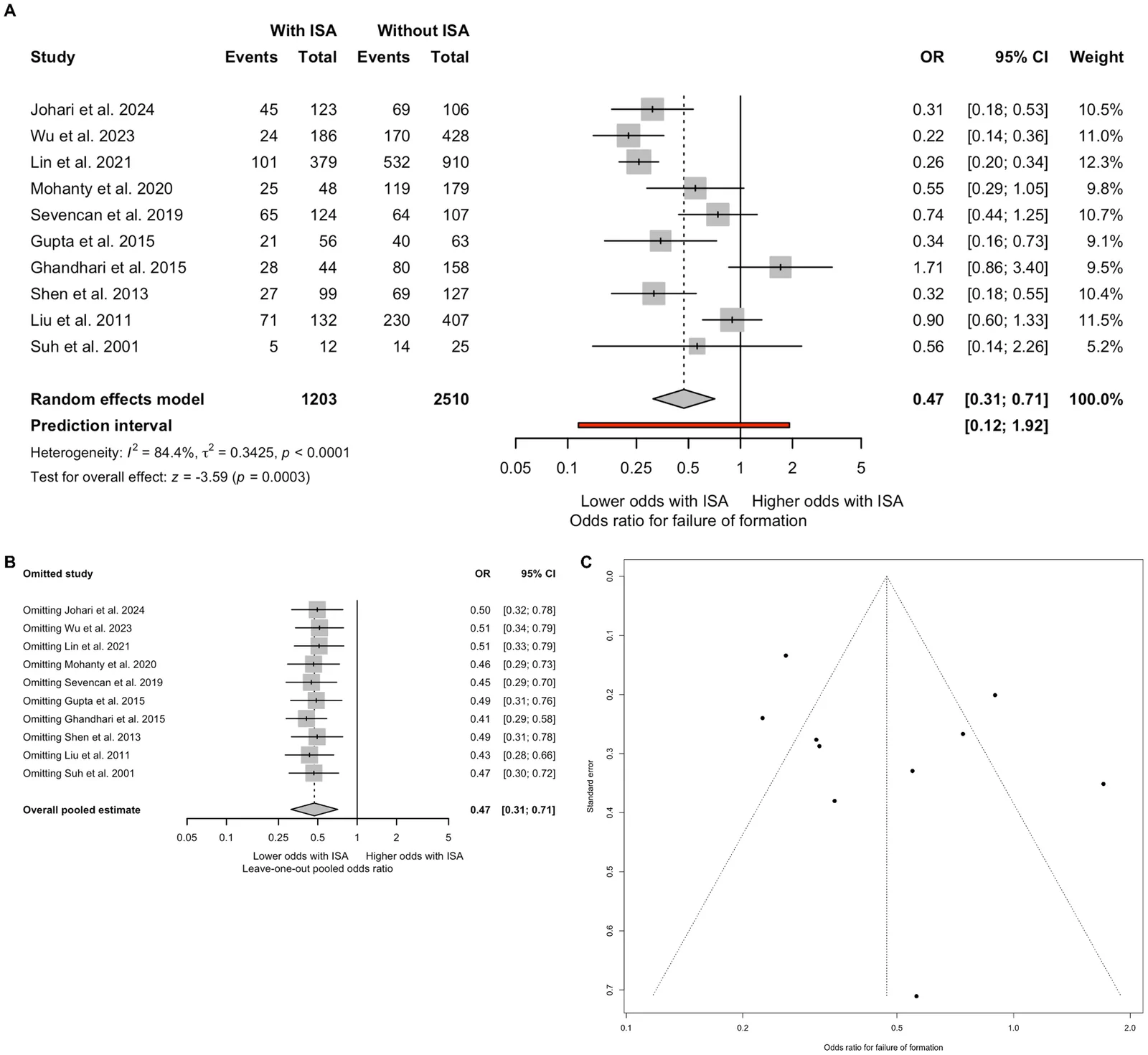
Failure of formation and intraspinal abnormalities across 10 studies comprising 3,713 patients, including 1,203 with and 2,510 without intraspinal abnormalities. (A) Forest plot comparing the odds of failure of formation in patients with versus without intraspinal abnormalities. (B) Leave-one-out sensitivity analysis. (C) Funnel plot. CI, confidence interval; ISA, intraspinal abnormalities; OR, odds ratio.

**Fig. 12:**
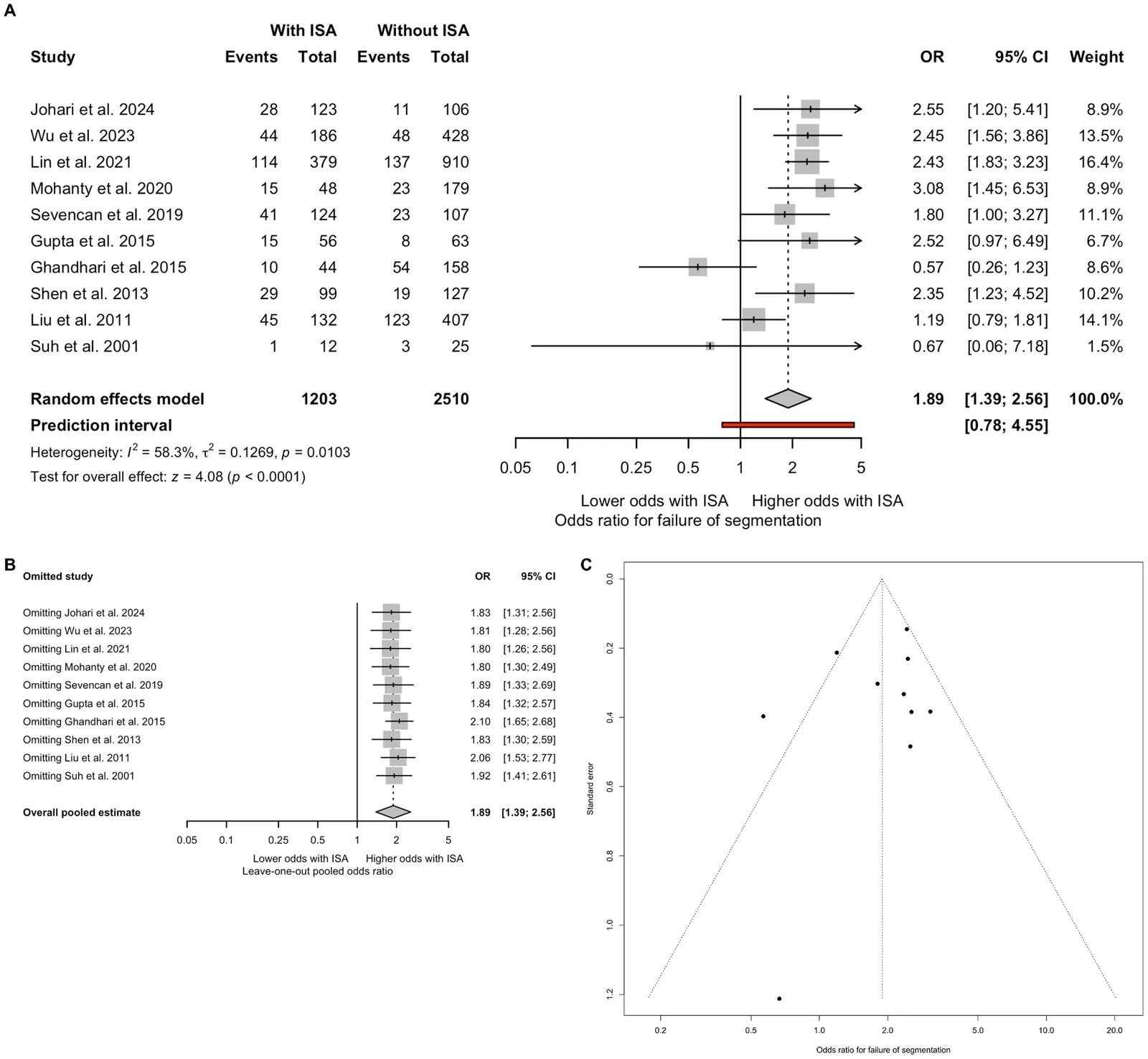
Failure of segmentation and intraspinal abnormalities across 10 studies comprising 3,713 patients, including 1,203 with and 2,510 without intraspinal abnormalities. (A) Forest plot comparing the odds of failure of segmentation in patients with versus without intraspinal abnormalities. (B) Leave-one-out sensitivity analysis. (C) Funnel plot. CI, confidence interval; ISA, intraspinal abnormalities; OR, odds ratio.

**Fig. 13:**
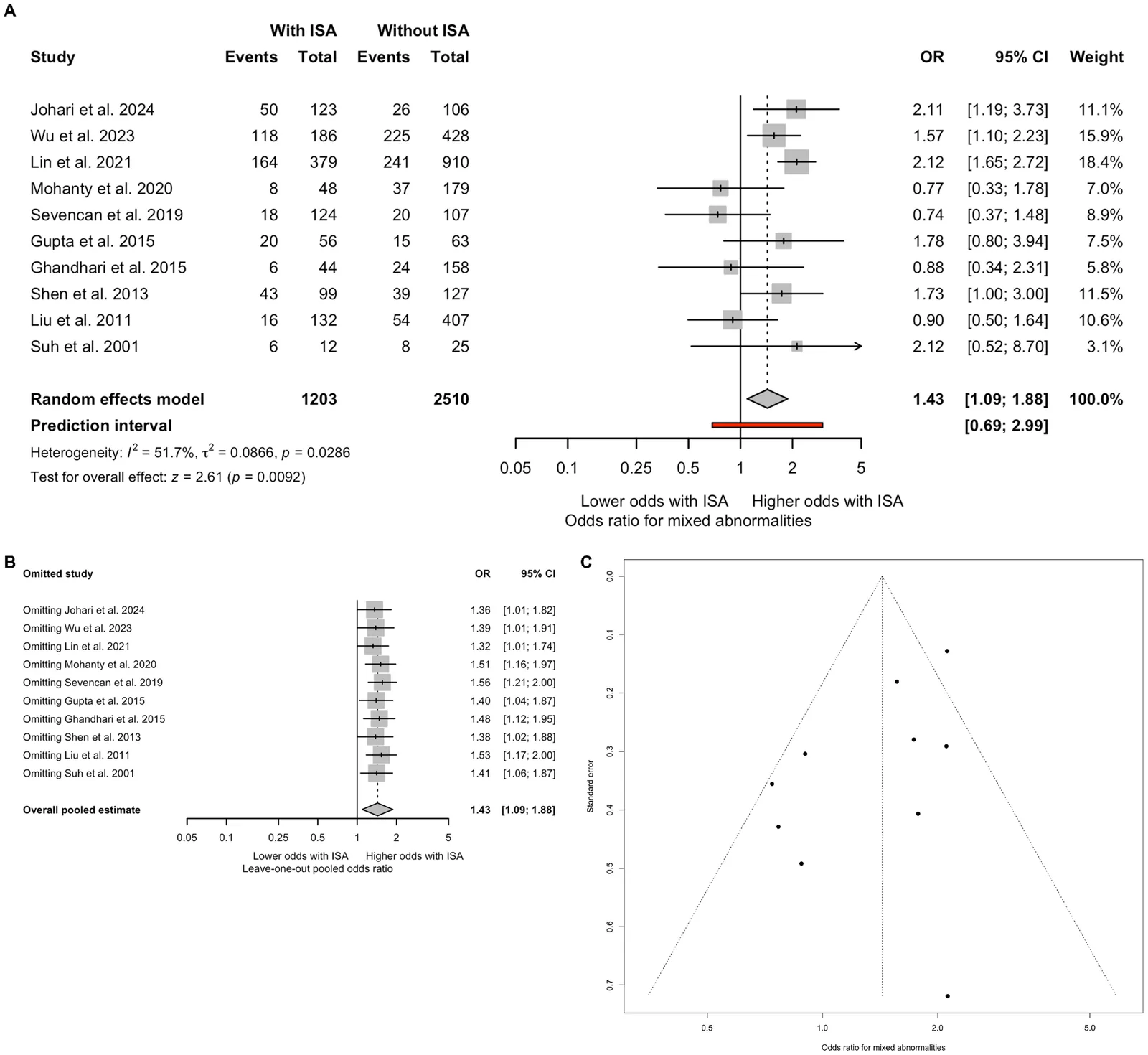
Mixed congenital scoliosis abnormalities and intraspinal abnormalities across 10 studies comprising 3,713 patients, including 1,203 with and 2,510 without intraspinal abnormalities. (A) Forest plot comparing the odds of mixed abnormalities in patients with versus without intraspinal abnormalities. (B) Leave-one-out sensitivity analysis. (C) Funnel plot. CI, confidence interval; ISA, intraspinal abnormalities; OR, odds ratio.

## Discussion

This updated systematic review and meta-analysis included 18 studies comprising 4,463 patients with CS. The pooled prevalence of intraspinal abnormalities was 35%, consistent with the 37% estimate reported in the previous meta-analysis of 10 studies and 1,863 patients [6]. Beyond confirming the overall prevalence, the expanded evidence base allowed evaluation of geographic variation, vertebral anomaly patterns, and Cobb angle. Sex-specific prevalence was 31% among male patients and 39% among female patients, comparable to the rates reported by Wang et al. of 30% and 41%, respectively [6]. These findings further support the absence of a significant association between patient sex and the presence of intraspinal abnormalities in CS.

Furthermore, subgroup analysis by geographical region showed that the Middle East had the highest reported prevalence of intraspinal abnormalities among patients with CS, at 37%, followed by South Asia and East Asia at 34%, and North America at 33%. No statistically significant subgroup difference was identified, and the similar pooled estimates suggest that marked geographic variation was not evident among the populations represented. To our knowledge, no previous study has directly compared regional differences in the prevalence of intraspinal abnormalities. However, geographic coverage was incomplete, with only 3 North American studies and no included cohorts from Europe, Africa, Latin America, or Australasia, and regional estimates may also have been influenced by differences in referral patterns, MRI indications, and cohort composition. Thus, the findings suggest broadly similar prevalence among the populations represented, but broader geographic generalization remains uncertain.

This meta-analysis showed that, among the 1,474 patients with intraspinal abnormalities, diastematomyelia was reported in 53.5%, followed by syringomyelia in 40.4% and tethered cord in 29.9%. This finding contrasts with previous reports in the literature, which identified tethered cord as the most common intraspinal abnormality among patients with CS [7,17,18]. Differences in diagnostic terminology and reporting across studies should be considered when comparing the frequencies of specific abnormalities.

Regarding vertebral anomalies, the current study showed that failure of segmentation and mixed vertebral anomalies were associated with a higher prevalence of intraspinal abnormalities, at 47% and 40%, respectively, compared with failure of formation, which showed a lower prevalence of 26%. This finding is consistent with previous studies reporting a stronger relationship between segmentation defects, mixed vertebral anomalies, and underlying intraspinal abnormalities [3,10,18]. Therefore, patients with CS who present with failure of segmentation or mixed vertebral anomalies may represent a higher-risk subgroup for intraspinal abnormalities, although these associations do not establish that vertebral anomaly pattern is an independent predictor. From a clinical perspective, this finding supports the importance of MRI evaluation, particularly before surgical intervention [5,7]. However, the prevalence of intraspinal abnormalities among patients with failure of formation also remained clinically relevant. Thus, vertebral anomaly pattern may inform risk assessment but should not be used in isolation to determine the need for MRI.

Cobb angle was higher among patients with intraspinal abnormalities than among those without. However, this difference was not statistically significant, and heterogeneity was substantial. Some individual cohorts reported larger Cobb angles in patients with intraspinal abnormalities [3,13]. In contrast, the findings of the present review suggest that curve magnitude alone may not reliably distinguish patients with and without intraspinal abnormalities. Therefore, Cobb angle should be considered alongside vertebral anomaly pattern, neurologic findings, age, and surgical context rather than used as an isolated criterion for MRI evaluation. One possible explanation for this discrepancy is the heterogeneity among the included studies, including differences in patient age, sample size, and the proportion of surgically treated patients.

This study has several strengths. It updates the prior evidence base by including more studies and a larger pooled sample, while also evaluating vertebral anomaly types, geographic region, publication era, and Cobb angle, providing a broader and more clinically relevant analysis. In addition, random-effects models, prediction intervals, and sensitivity analyses were used to account for between-study variability and assess the robustness of the pooled estimates. However, this study also has limitations. Substantial heterogeneity remained despite subgroup analyses and may reflect differences in MRI protocols, age distributions, referral patterns, surgical versus nonsurgical cohort composition, single- versus multicenter study design, and definitions of CS and individual intraspinal abnormalities across studies. MRI indications were also not consistently reported, and selective imaging based on neurologic findings, planned surgery, deformity severity, or other clinical concerns cannot be excluded. These factors may have influenced the reported prevalence of intraspinal abnormalities and may limit generalizability to unselected patients with newly diagnosed CS. The vertebral anomaly comparisons were based on unadjusted study-level data and could not account for potential confounding by age, deformity characteristics, referral patterns, or MRI practices. Despite this heterogeneity, leave-one-out sensitivity analyses showed stable pooled estimates, suggesting that the overall result was not driven by a single study. Cobb angle analyses were limited by the small number of studies with complete group-specific data.

The included studies did not consistently distinguish symptomatic from incidentally detected intraspinal abnormalities, precluding separate analysis by symptom status. Future studies should report this distinction more systematically to better define the clinical relevance of MRI-detected abnormalities for surgical planning. Future studies with standardized methods and consistent reporting are needed to improve the reliability and comparability of findings.

## Conclusion

In conclusion, this meta-analysis demonstrated that intraspinal abnormalities are relatively common in MRI-evaluated cohorts of patients with CS, with an overall pooled prevalence of 35%. Failure of segmentation and mixed vertebral anomalies were associated with a higher prevalence of intraspinal abnormalities in unadjusted study-level analyses, suggesting that these patients may represent a higher-risk subgroup warranting greater clinical suspicion, although vertebral anomaly pattern alone should not determine MRI use.

## Data availability

The data used during the current study are available from the corresponding author upon reasonable request.

## CRediT authorship contributions

**Nizar Algarni:** Supervision, Methodology, Writing - Review & Editing. **Khalid Alrasheed:** Methodology, Formal analysis, Investigation, Data Curation, Writing - Original Draft, Writing - Review & Editing, Visualization, Project administration. **Faisal Alkhunein:** Conceptualization, Investigation, Data Curation, Writing - Original Draft, Writing - Review & Editing, Project administration. **Mohammed Alorayyidh:** Data Curation, Investigation, Writing - Original Draft, Writing - Review & Editing. **Rahaf Alrasheed:** Data Curation, Investigation, Writing - Original Draft, Writing - Review & Editing. **Yousef Marwan:** Supervision, Methodology, Writing - Review & Editing.

## Funding

This research did not receive any specific grant from funding agencies in the public, commercial, or not-for-profit sectors.

## Declaration of competing interests

The authors declared no potential conflicts of interest with respect to the research, authorship, and/or publication of this article.

## Appendix Supplementary materials

Image, application 1
