# Supplementary material for: Intraspinal abnormalities in MRI-evaluated congenital scoliosis: a systematic review and meta-analysis of prevalence, geographic variation, vertebral anomaly patterns, and Cobb angle
Source: N Am Spine Soc J. 2026 Sep 12;28:100943. doi: 10.1016/j.xnsj.2026.100943 (PMC13693074; doi:10.1016/j.xnsj.2026.100943)
Supplement: Supplementary file 1 [file mmc1.docx]

**Supplementary tables**

**Table S1**. Search strategy

| **Database** | **Search strategy** |
| --- | --- |
| Pubmed/MEDLINE | (MRI[Title/Abstract] OR magnetic[Title/Abstract] OR resonance[Title/Abstract] OR imaging[Title/Abstract]) AND congenital[Title/Abstract] AND scoliosis[Title/Abstract] |
| Web of Science | TS=((MRI OR magnetic OR resonance OR imaging) AND congenital AND scoliosis) |
| Scopus | TITLE-ABS-KEY((MRI OR magnetic OR resonance OR imaging) AND congenital AND scoliosis) |
| Cochrane | (congenital):ti,ab,kw AND (scoliosis):ti,ab,kw AND (MRI OR magnetic OR resonance OR imaging):ti,ab,kw |

**Table S2**: MINORS Assessment Tool for Non-randomized Studies (n = 18)

| **Item** | **Clearly stated aim** | **Inclusion of consecutive patients** | **Prospective data collection** | **Endpoints appropriate to the study aim** | **Unbiased assessment of the study endpoint** | **Follow-up period appropriate to the study aim** | **Loss to follow-up less than 5%** | **Prospective calculation of the study size** | **An adequate control group** | **Contemporary groups** | **Baseline equivalence of groups** | **Adequate statistical analyses** |
| --- | --- | --- | --- | --- | --- | --- | --- | --- | --- | --- | --- | --- |
| Alkhunein et al. 2026 [16] | 2 | 2 | 0 | 2 | 1 | 2 | 0 | 0 | NA | NA | NA | NA |
| Khan et al. 2025 [17] | 2 | 1 | 0 | 2 | 2 | 2 | 0 | 1 | NA | NA | NA | NA |
| Johari et al. 2024 [13] | 2 | 1 | 0 | 2 | 1 | 2 | 0 | 0 | NA | NA | NA | NA |
| Wu et al. 2023[3] | 2 | 2 | 0 | 2 | 1 | 2 | 1 | 0 | NA | NA | NA | NA |
| Lin et al. (I/II) 2021[11,12] | 2 | 2 | 0 | 2 | 1 | 2 | 1 | 0 | NA | NA | NA | NA |
| Yarandi et al. 2021[18] | 2 | 1 | 0 | 2 | 1 | 2 | 2 | 0 | NA | NA | NA | NA |
| Mohanty et al. 2020 [5] | 2 | 2 | 0 | 2 | 1 | 1 | 1 | 0 | NA | NA | NA | NA |
| Furdock et al. 2019[4] | 2 | 1 | 0 | 2 | 1 | 2 | 0 | 0 | NA | NA | NA | NA |
| Sevencan et al. 2019 [9] | 2 | 1 | 0 | 2 | 1 | 2 | 0 | 0 | NA | NA | NA | NA |
| Hou et al. 2018 [19] | 2 | 2 | 0 | 2 | 1 | 2 | 1 | 0 | 1 | 2 | 1 | 1 |
| Gupta et al. 2015 [20] | 2 | 2 | 0 | 2 | 1 | 2 | 0 | 0 | NA | NA | NA | NA |
| Ghandhari et al. 2015 [21] | 2 | 2 | 0 | 2 | 1 | 2 | 0 | 0 | 2 | 2 | 1 | 1 |
| Shen et al. 2013 [10] | 2 | 2 | 0 | 2 | 1 | 0 | 0 | 0 | NA | NA | NA | NA |
| Liu et al. 2011 [22] | 2 | 2 | 0 | 2 | 1 | 0 | 0 | 0 | NA | NA | NA | NA |
| Rajasekaran et al. 2010 [7] | 2 | 2 | 2 | 2 | 1 | 0 | 0 | 0 | NA | NA | NA | NA |
| Erfani et al. 2007 [23] | 2 | 1 | 0 | 2 | 1 | 2 | 0 | 0 | NA | NA | NA | NA |
| Belmont et al. 2004 [24] | 2 | 2 | 0 | 2 | 1 | 2 | 0 | 0 | NA | NA | NA | NA |
| Suh et al. 2001 [25] | 2 | 1 | 0 | 2 | 1 | 2 | 0 | 0 | NA | NA | NA | NA |
